# Molecular Mechanisms of Drug Resistance and Epidemiology of Multidrug-Resistant Variants of *Neisseria gonorrhoeae*

**DOI:** 10.3390/ijms231810499

**Published:** 2022-09-10

**Authors:** Beata Mlynarczyk-Bonikowska, Cezary Kowalewski, Aneta Krolak-Ulinska, Wojciech Marusza

**Affiliations:** 1 Department of Dermatology, Immunodermatology and Venereology, Medical University of Warsaw, Koszykowa 82a, 02-008 Warsaw, Poland; 2 Academy of Face Sculpting, Jana Kazimierza 11B, 01-248 Warsaw, Poland

**Keywords:** *Neisseria gonorrhoeae*, mechanisms of resistance, NG-MAST, NG-STAR, MLST, treatment

## Abstract

The paper presents various issues related to the increasing drug resistance of *Neisseria gonorrhoeae* and the occurrence and spread of multidrug-resistant clones. One of the most important is the incidence and evolution of resistance mechanisms of *N. gonorrhoeae* to beta-lactam antibiotics. Chromosomal resistance to penicillins and oxyimino-cephalosporins and plasmid resistance to penicillins are discussed. Chromosomal resistance is associated with the presence of mutations in the PBP2 protein, containing mosaic variants and nonmosaic amino acid substitutions in the transpeptidase domain, and their correlation with mutations in the *mtrR* gene and its promoter regions (the MtrCDE membrane pump repressor) and in several other genes, which together determine reduced sensitivity or resistance to ceftriaxone and cefixime. Plasmid resistance to penicillins results from the production of beta-lactamases. There are different types of beta-lactamases as well as penicillinase plasmids. In addition to resistance to beta-lactam antibiotics, the paper covers the mechanisms and occurrence of resistance to macrolides (azithromycin), fluoroquinolones and some other antibiotics. Moreover, the most important epidemiological types of multidrug-resistant *N. gonorrhoeae*, prevalent in specific years and regions, are discussed. Epidemiological types are defined as sequence types, clonal complexes and genogroups obtained by various typing systems such as NG-STAR, NG-MAST and MLST. New perspectives on the treatment of *N. gonorrhoeae* infections are also presented, including new drugs active against multidrug-resistant strains.

## 1. Introduction

The increasing antimicrobial drug resistance of *Neisseria gonorrhoeae*, the etiologic agent of gonorrhea, is becoming a global problem. Gonorrhea is among the most common bacterial sexually transmitted infections, with the WHO estimating in 2020 that 82,400,000 cases of the disease occurred worldwide [1]. In addition, the incidence of reported cases of gonorrhea in the European Union is increasing. Per 100,000 people in the population, an increase was observed from 6.6 in 2009 to 26.9 in 2018 [2,3]. According to the Centers for Disease Control and Prevention (CDC) in the United States, the incidence of gonorrhea increased by 111% from 2009 to 2020. In 2019, the number of cases per 100,000 people was 187.8 and in 2020, 206.5 [4]. The susceptibility of *N. gonorrhoeae* to drugs (ceftriaxone, cefixime, azithromycin, ciprofloxacin, spectinomycin and gentamicin) in EU countries and the UK is monitored on an ongoing basis as part of the European Gonococcal Antimicrobial Susceptibility Programme (Euro-GASP), which is funded by the European Centre for Disease Prevention and Control (ECDC) [5,6,7,8]. The antibiotic resistance of *N. gonorrhoeae* is monitored in the United States as part of the GISP (Gonococcal Isolate Surveillance Project), funded by the CDC.

In 2017, *N. gonorrhoeae* was included in a WHO-created list of 12 pathogens whose drug resistance poses a global health threat and which most urgently require the development of new antibiotics due to the worldwide emergence of multidrug-resistant strains including those resistant to beta-lactams (ceftriaxone, cefixime and penicillin) and simultaneously resistant to oxyimino-cephalosporins (ceftriaxone, cefixime) and azithromycin [9]. In recent years, *N. gonorrhoeae* strains collected from various EU countries, including Poland, have been characterized by the Euro-GASP for antibiotic susceptibility, which was studied using conventional methods, and their genomes were sequenced using the WGS (whole-genome sequencing) method. The WGS method makes it possible to specify the genetic determinants of resistance and replaces other methods of bacteria molecular typing, allowing simultaneous determination of membership in such sequence types as NG-MAST, NG-STAR and MLST. The results of these studies, depicting the epidemiological situation in the countries studied, have been published [5,6,10]. Sequences of newly described determinants of resistance to antibiotics and new epidemiological types are constantly updated in the worldwide NG-STAR, NG-MAST and MLST databases.

## 2. Resistance to Beta-Lactam Antibiotics

**Figure 1:**
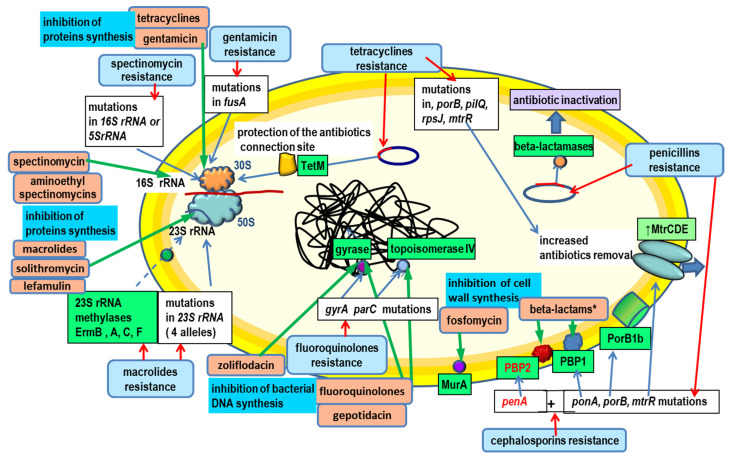
The most important targets of antibiotics (green arrows) and mechanisms of resistance (red arrows) in *N. gonorrhoeae.* See text for details. Beta-lactams*: penicillins, cephalosporins (ceftriaxone, cefixime), carbapenems (ertapenem).

Beta-lactams act on certain enzymes (transpeptidases, transglycosylases, carboxypeptidases) involved in the synthesis of peptidoglycan, a key component of the bacterial cell wall. Proteins inactivated by beta-lactams are referred to as PBPs (penicillin-binding proteins). There are four PBPs in *N. gonorrhoeae*. The two high-molecular-weight PBPs, PBP1 (class A) and PBP2 (class B), play a key role in beta-lactam resistance. Both are characterized by their high molecular weight. The other two proteins, PBP3 and PBP4, which are characterized by a low molecular weight and exhibit both carboxypeptidase and endopeptidase activities, are not important in the acquisition of beta-lactam resistance in *N. gonorrhoeae.* Inactivation of specific PBPs leads to bacterial cell death. The targets of action of beta-lactam antibiotics are shown in Figure 1.

### 2.1. Resistance to Oxyimino-Cephalosporins (Ceftriaxone and Cefixime)

The resistance criteria for ceftriaxone, cefixime and cefotaxime according to the EUCAST is MIC > 0.125 mg/L [11]. The CLSI [12] has not established resistance criteria for ceftriaxone and cefixime, but provides susceptibility criteria for these antibiotics (MIC ≤ 0.25 mg/L). *N. gonorrhoeae* strains with a ceftriaxone MIC of 0.25 mg/L were isolated in Canada as early as 1982–84 [13]. *N. gonorrhoeae* strains with a ceftriaxone MIC of 0.5 mg/L have been appearing sporadically on various continents for a long time, but did not spread until 2015. Five such strains have been described in the US, with the first one isolated as early as 1987 in San Diego (California), another two such strains in 1992 and 1993 in Cincinnati (Ohio), another in 1997 in Philadelphia, (Pennsylvania) and another in 2012 in Oklahoma City (Oklahoma) [14,15]. The first *N. gonorrhoeae* strain resistant to ceftriaxone (MIC = 0.5 mg/L) found in multiple countries and continents was FC428 isolated in Japan in 2015 [3,16,17,18,19]. The characteristics of FC428 are shown in Table 1.

In recent years, *N. gonorrhoeae* strains with a ceftriaxone MIC ≥ 0.5 mg/L have been most frequently described in Asian countries [20,21,22,23,24,25,26]. Among 259 *N. gonorrhoeae* strains isolated in 2017–20 in China, 9 (3.5%) were resistant to ceftriaxone (MIC ≥ 0.5 mg/L) and cefixime (MIC ≥ 2 mg/L). The ceftriaxone MIC range for these 259 isolates was 0.06 to 1 mg/L (MIC50, 0.125 mg/L; MIC90, 0.125 mg/L) and the cefixime MIC range was 0.06 to ≥4 mg/L (MIC50, 0.125 mg/L; MIC90, 0.5 mg/L) [26]. Among the 2375 *N. gonorrhoeae* strains isolated in 26 European countries in 2018, 1 ceftriaxone-resistant strain (MIC = 0.5 mg/L) similar to FC428 and 36 cefixime-resistant strains (MICs 0.25–2 mg/L) were found [27]. All 36 cefixime-resistant isolates had mosaic mutations in PenA: 25 isolates NG-STAR PenA allele: 10.001 (MICs 0.25–0.50 mg/L); 9 isolates NG-STAR PenA allele: 34.001 (MIC = 0.25 mg/L); 1 isolate NG-STAR PenA allele: 60.001 (MIC = 2 mg/L); and 1 NG-STAR PenA allele: 171.001 (MIC = 0.25 mg/L) [27]. In Europe, a decline in the incidence of ceftriaxone-resistant *N. gonorrhoeae* strains has been observed since 2009 [3]. This trend may have been due in part to a treatment change introduced by the 2012 European gonorrhea guidelines, in which cefixime 400 mg was replaced by ceftriaxone 500 mg plus azithromycin 2 g in dual therapy as the recommended empiric first-line treatment for uncomplicated gonorrhea. In 2020, the recommended first-line treatment was refined to ceftriaxone 1 *g* plus azithromycin 2 g or ceftriaxone 1 g monotherapy in well-controlled settings where there is no resistance to ceftriaxone [3,28]. Internationally isolated *N. gonorrhoeae* strains with ceftriaxone MICs ≥ 0.5 mg/L are shown in Table 1 [17,18,19,20,21,22,23,24,25,27,28,29,30,31,32,33,34,35,36,37,38,39,40,41,42,43,44,45,46].

Crucial in the development of *N. gonorrhoeae* resistance to ceftriaxone and cefixime are mutations in the *penA* gene, especially in the domain encoding PBP2 transpeptidase. An increase in the MICs of these drugs can also result from the overproduction of MtrCDE membrane pump proteins (most commonly a deletion of −35A) in the *mtrR* promoter region, and substitutions in the MtrR protein (G45D) and amino acid substitutions in the PorB1b protein, but both of these mechanisms alone do not determine resistance [47]. Although the link between *N. gonorrhoeae* resistance to oxyimino-cephalosporins and the above-mentioned mutations is indisputable, strains with identical or very similar genetic characteristics but drastically different MICs of cephalosporins have been isolated [48,49,50,51,52]. The implication is that some factor determining resistance to ceftriaxone and cefixime is being missed, and it is impossible to predict from DNA sequencing alone whether *N. gonorrhoeae* is resistant to cefixime and ceftriaxone. This may involve regulatory mechanisms regarding PBP2 protein expression, which translates into the amount of PBP2 protein in the *N. gonorrhoeae* cell. Mechanisms of resistance to beta-lactams resulting from the overproduction of PBP proteins have been described in Gram-positive bacteria [53]. The effect of certain mutations in the *rpoB* and *rpoD* genes (they encode DNA-dependent RNA polymerase subunits) on changes in the MIC and the development of resistance to ceftriaxone and cefixime is presented in Table 2 [54].However, the mechanism of this resistance is not currently understood.

Mutations in the *penA* gene encode PBP2 transpeptidase. Based on sequencing of a 1745–1752 bp fragment of the *penA* gene, about 489 alleles of the PBP2 protein have been described, which are divided into 234 major types (I-V, VII, IX-XIX, XXI-XXII, XXVII, XXXIV-XXXV, 37-234) and many subtypes (Table 3 and Table 4).The proteins encoded by the individual alleles contain from 1 to over 60 amino acid mutations compared to ancestral wild-type alleles. Mutation-induced changes in the amino acid pattern of PBP2 transpeptidase (mostly substitutions and single amino acid deletions and insertions), are referred to as mosaic, semimosaic or nonmosaic patterns of PBP2. The mosaic-like structure of the *penA* gene in *N. gonorrhoeae* has evolved by intragenic recombination with *penA* genes of other *Neisseria* species, which are mainly commensal: *Neisseria perflava*, *Neisseria sicca*, *Neisseria cinerea*, *Neisseria flavescens* and Neisseria meningitidis. Mosaic alleles of PBP2 have approximately 60 amino acid alterations from the PBP2 of wild-type strains and most often the D346a insertion is absent [55,56]. Semimosaic alleles usually have 20–30 amino acid alterations and some have the D346a insertion [57]. The nonmosaic alleles usually have a D346a insertion and between 1 and 13 (most often 4–8) mutations in the C-terminus [57]. In mosaic patterns, the most important substitutions responsible for ceftriaxone and cefixime resistance (MIC > 0.125 mg/L) are A311V, I312M, V316P/T, T483S, A501P/V/T/T, F504L, N512Y A516G and G545S [40,47,57,58,59,60], and in nonmosaic patterns, the substitutions are A501P/V/T/R/S, F504L, N512Y, A516G, G542S and P551S [47,57,58,59,61,62]. Moreover, A516G does not appear to alter resistance very much [62]. The main types of nonmosaic, semimosaic and mosaic PenA are shown in Table 3 and Table 4 [30,40,41,42,43,44,52,54,57,63,64,65]. There are differences in the numbering of some amino acids (usually by 1) between the source papers and the NG-STAR databases.

Tomberg et al. showed that the transformation of FA19 (an antibiotic-susceptible strain) with the WHO X *penA* allele is sufficient to confer resistance to ceftriaxone and cefixime that surpasses the EUCAST- and CDC-defined resistance (MICs for ceftriaxone = 0.31 mg/L and for cefixime = 1.6 mg/L) [59]. However, the presence of a mosaic *penA* allele alone in most cases is not sufficient for the resistance to these antibiotics and other determinants, as substitutions in the proteins PonA (L421P) and PorB, mutations in the promoter region of the *mtrR* gene (most often deletion −35A) and *mtrCDE*, and/or substitutions in the MtrR protein (G45D) are needed [20,23,66,67].

The MtrCDE membrane pump removes beta-lactams, macrolides, tetracyclines, rifampicin and detergents. The expression of *mtrCDE* in wild-type gonococci is repressed by MtrR and, in the presence of an inductor, is activated by MtrA. MtrA and MtrR bind to regions within a 250 bp sequence that contains overlapping, divergent promoters for the transcription of *mtrR* and *mtrCDE*. The overproduction of membrane pump proteins is determined by substitutions in the MtrR pump repressor protein (G45D and A39T) and mutations in the region upstream of the *mtrR* gene (e.g., deletion of adenine at the −35 position preceding the *mtrR* gene and mutation conditioning the formation of a new *mtrCDE* promoter, the so-called *mtr120*, which is not regulated by MtrR and MtrA) [68,69,70,71,72,73]. The expression and efficiency of the MtrCDE pump are also significantly influenced by mosaic changes within the *mtrR* and *mtrCDE* genes, probably caused by the transformation and recombination of DNA from saprophytic *Neisseria* spp. (e.g., *N. lactamica*) and *N. meningitidis*. Mosaic sequences within the gene encoding the inner membrane transporter protein *mtrD* have shown a strong linkage and epistatic effects that likely enhance the efflux pump activity of MtrCDE [70,73]. Alterations within the *mtrR* and *mtrCDE* genes, especially mosaic alterations within *mtrD*, are one of the most important determinants in *N. gonorrhoeae* of a novel azithromycin resistance phenotype (MIC of 2 to 4 mg/L), which is not associated with *23S rRNA* mutations (for details, see Section 3) [70,73].

Amino acid substitutions of the PorB1b protein at positions 120 and 121 result in changes in the MIC of penicillins, cephalosporins and tetracyclines. Based on the sequencing of a 30 bp fragment of PorB1b, the amino acid substitutions of the PorB1b protein were determined to be G120C, D, E, K, N, P, Q, R, S or T and A121D, G, H, N, P, S or V [71]. Mutations in the *porB* gene determining substitutions in the PorB1b protein (G120K, G120D/A121D, G120P/A121P and G120R/A121H) are named *penB* mutations [73,74,75]. *PorB* mutations reduce antibiotic diffusion into the periplasmic space only in the presence of *mtrR* or *mtr* promoter mutations [73]. The combined occurrence of mutations in the *porB* and *mtr* genes in strain FA19penA4 (penA4 being the allele from FA6140, a pen-resistant isolate) increased the MIC of penicillin from 0.12 mg/L to 1.0 mg/L and the MIC of tetracycline from 0.15 mg/L to 1.0 mg/L. The combined presence of mutations in the *porB* and *mtr* genes and the *pilQ* mutation (named the *penC* mutation) in this strain increased the MIC of penicillin to 2.0 mg/L and the MIC of tetracycline to 2.0 mg/L, whereas the simultaneous occurrence of four mutations in the *porB, mtr*, *ponA* (L429P in the PonA protein) and *pilQ* genes increased the MIC of penicillin to 4.0 mg/L [74].Together, the PilQ protein and type IV pili form an SDS-resistant multimeric complex that acts as a pore for antibiotics and small molecules. A mutation causing a G666L substitution in the PilQ protein, known as the *pilQ2* or *penC* mutation, hinders the formation of the PilQ multimer, disrupting pore formation and thereby reducing penicillin influx [76]. The *pilQ* mutations in these studies arise spontaneously and at a high frequency in the laboratory. However, no study has found that *pilQ* mutations are present in clinical isolates, likely because the bacteria require an intact type IV pilus machinery for infection [77].

### 2.2. Penicillin Resistance

The cause of resistance to benzylpenicillin (MIC > 1 mg/L according to the EUCAST [11] and MIC ≥ 2 mg/L according to the CLSI [12]) is mutations in specified chromosomal genes or the production of beta-lactamase. The following genes play the most important role in chromosomal resistance to penicillin: genes determining the overproduction of MtrCDE proteins [66,67,78,79,80], as well as *penA* (substitutions in PenA: I312M, V316P/T, ins346D, T483S, A501P/T/V, G542S, G545S, P551S) [40,58,59], *porB1b* (substitutions in PorB1bG120K/A121N/D) *pilQ* (G666L substitution in the PilQ protein)and *ponA* (substitution in PonA L421P) [72,74] (Figure 1). The combinations of mentioned substitutions may determine the resistance to penicillin (MIC 2 − 4 mg/L).

The second major reason for penicillin resistance in *N. gonorrhoeae* is the production of plasmid-encoded beta-lactamases: TEM-1 (most common), TEM-1B, TEM-135 (M182T substitution), TEM-220 (M182T and A185T substitutions), TEM-141 (K34E substitution), TEM-198 (T271I substitution) and new alleles that are constantly being described [81,82,83,84,85,86]. Thus far, seven types of penicillinase plasmids have been described: Asia (7405–7428 bp; pJD4 prototype; beta-lactamase TEM-1 or TEM-135; GenBank NC_002098, U20374-U20375, NZ_MK973072-NZ_MK973081) [87,88], Africa (5597–5601 bp; pJD5 prototype; Asia plasmid with deletion from 1880 bp to 3708 bp; beta-lactamase TEM-1 or TEM135; GenBank acc. NZ_MK973082-NZ_MK973086 and NZ_MH140435) [89,90], Toronto/Rio (5154–5161 bp; pSJ5.2, pJD7 and pGo4717 prototypes; Asia plasmid with deletion from 3802 to 6075 bp; beta-lactamase TEM-135, TEM-1 or TEM220; GenBank acc. NC_010881, DQ355980; U20419) [91], Nimes (6798 bp; pGF1 prototype; Asia plasmid with deletion from 1880 to 3708 and IS5 insertion of 1200 bp between 604 and 605 nucleotides; GenBank acc. U20421) [87], New Zealand (9309 bp; pAS84/417 prototype; Asia plasmid, tandem repeat duplication 1883 bp from nucleotide 593; GenBank U20422) [87], Johannesburg (4865 bp; pEM1 prototype; Asia plasmid with two deletions 1928–4487 and 6236; beta-lactamase TEM1; GenBank acc. HM756641, NC_019211) [92], and Australia (3269 bp; Asia plasmid with two deletions 502-2385 and 3795-6066 and transition T7424C; beta-lactamase TEM-135; GenBank acc. NC_025191, KJ842484) [93]. The prevalence of penicillinase-producing strains (PPNGs) varies in different years and according to geographic region. High percentages of PPNGs were found in some Asian countries: Thailand (84% of PPNGs), Sri Lanka (63.6% of PPNGs), India (45% of PPNGs), Myanmar (35% of PNPGs) and Bhutan (88.9% of PPNGs) in isolated strains in 2009–2012 [94]. In China, PPNGs account for 41% in 2011–2012 and 2015–2017 [89].

TEM beta-lactamases are common in bacteria. In total, 197 (numbering from 1 to 246, some numbers removed) types of these enzymes have been described, and enzymes with an extended spectrum that hydrolyze oxyimino-cephalosporins, i.e., ceftriaxone and cefixime, among others, are commonly used in the treatment of gonorrhea [95,96]. It is only a matter of time before such TEM variants appear in *N. gonorrhoeae*. It is difficult to predict whether these will be sporadic incidents or whether a strain with such an enzyme variant is likely to spread endemically or pandemically in the population.

## 3. Macrolide (Azithromycin) Resistance

Azithromycin binds to a four-nucleotide fragment of rRNA (in the peptidyltransferase region) within the V domain of the 23S rRNA in the 50S subunit of the ribosome, which results in the inhibition of protein synthesis (Figure 1). The azithromycin resistance criterion according to the EUCAST is an MIC > 1.0 mg/L [11]. The CLSI only gives a criterion for azithromycin susceptibility (MIC ≤ 1.0 mg/L) [12]. Transition of A2059G (*Escherichia coli* numbering) in all four alleles of the 23S rRNA gene determines resistance and an MIC of azithromycin > 256 mg/L. Transition of C2611T or transversion of C2611G in all four alleles of the *23S rRNA* gene determines resistance and an MIC of azithromycin that is 2–32 mg/L [97,98,99] (Figure 1).

In addition, the overproduction of MtrCDE proteins is usually caused by mutations in the promoter region of the *mtrR* gene, which is most often −35A, and within the *mtrR* gene, which is most often a substitution in the MtrR protein G45D [99,100]. Substitutions in the MtrR protein of G45S, A86T and Y105H have also been described, conditioning azithromycin resistance (MIC 2–8 mg/L) in some strains, as well as substitutions of D79N, A39T, L99G or H which increased the MIC value, but most often did not cause resistance [68]. The cited paper lacks complete molecular characterization of strains in which azithromycin resistance or elevated MICs were detected; therefore, substitutions in MtrR may not be related to azithromycin MIC changes and the cause may be different. The cause of azithromycin MIC changes may also be mosaic changes in *mtrR* and/or *mtrD* resulting from the transformation and recombination with DNA from other *Neisseria* species [70]. Furthermore, also described is *N. gonorrhoeae* strain FA19 (*mtrR*, wt; *mtrR* promoter/*mtrCDE* promoter, wt; *mtrD*, wt; azithromycin MIC = 0.5 mg/L) and five variants of this strain: CR.100 (MtrR, D79N, S183N, M197I; *mtrR* promoter/*mtrCDE* promoter, -C35T/-T35G; *mtrD*, wt; azithromycin MIC = 1.0 mg/L), CR.101 (*mtrR*, wt; *mtrR* promoter/*mtrCDE* promoter, -C35T/-T35G; *mtrD*, wt; azithromycin MIC = 1.0 mg/L), CR.102 (MtrR, D79N; *mtrR* promoter/*mtrCDE* promoter, wt/wt; *mtrD*, wt; azithromycin MIC = 0.5 mg/L), CR.103 (MtrR, D79N, S183N, M197I; *mtrR* promoter/*mtrCDE* promoter, -C35T/-T35G; *mtrD*, 3-end mosaic-like; azithromycin MIC = 2.0 mg/L) and CR.104 (MtrR, D79N, S183N, M197I; *mtrR* promoter/mtrCDE promoter, -C35T/-T35G; MtrD, S821A, K823E; azithromycin MIC = 2.0 mg/L). Mutations in the *mtrR* promoter/*mtrCDE* promoter regions and in the *mtrD* gene have been shown to have the greatest impact on the increase in azithromycin MIC, and that the described mutations in the *mtrD* gene increase the efficiency of the *mtrCDE* pump relative to azithromycin [69]. A group of 107 *N. gonorrhoeae* isolates were also described (no mutations in 23S rRNA; *mtrR* mosaic; *mtrR* promoter C substitution; MtrD K823E; MLST: ST9363 and ST11422), among which 42 isolates had an MIC = 2 mg/L and 1 isolate had an MIC = 4 mg/L [101].

Mutations in the *rplV* genes that condition tandem duplications of ARAK at position 90 or KGPSLK at position 83 in ribosomal protein L22 [102] and mutations in the *rplD* gene that condition a G70D substitution in ribosomal protein L4 may also be the cause of the azithromycin MIC increase. This mutation causes an increase in the MIC of azithromycin 2.5-4-fold to a level of 0.4–0.5 mg/L, which does not cause azithromycin resistance according to current criteria [103].

Mutations in the promoter region of the *macA–macB* efflux system may also be the cause of the azithromycin MIC increase [104].

ErmB and ErmF 23S rRNA methylases were also detected in *N. gonorrhoeae* (Figure 1). The MIC of azithromycin in these strains had values ranging from 1 to 4 mg/L [105]. The lack of complete molecular characterization of these strains makes it impossible to determine what caused azithromycin resistance in thes e strains. The presence of *ermB*, *ermC*, *ermF* and *mefA* genes (encoding a membrane pump protein categorized as MFS) in many *N. gonorrhoeae* strains isolated between 1940 and 1987 has also been described [106]. The occurrence of different *erm* and *mefA* genes is characteristic for Gram-positive cocci and for Gram-negative anaerobic bacilli *Bacteroides* spp. [106,107].

In recent years, a successive increase in the percentage of azithromycin-resistant *N. gonorrhoeae* strains has been observed in many European countries [108].

## 4. Resistance to Fluoroquinolones (Ciprofloxacin)

Fluoroquinolones are classified as bactericidal drugs. They inhibit the activity of topoisomerase II (gyrase) and topoisomerase IV enzymes responsible for DNA supercoiling and relaxation (Figure 1). The resistance criteria for *N. gonorrhoeae* according to the EUCAST for ciprofloxacin is an MIC > 0.06 mg/L and for ofloxacin is an MIC > 0.25 mg/L [11]. According to the CLSI, the criterion for *N. gonorrhoeae* resistance to ciprofloxacin is an MIC ≥ 1.0 mg/L [12].

The cause of resistance to fluoroquinolones (most commonly to ciprofloxacin) are cumulative mutations in the QRDR (quinolone resistance-determining region) of *gyrA* (DNA gyrase subunit A, EC5.99.1.3), *parC* (DNA topoisomerase IV subunit A), and sometimes, *parE* (DNA topoisomerase IV subunit B, EC5. 99.1), causing amino acid substitutions in topoisomerase II and IV proteins: GyrA (S91F or T; D95A, G, N or Y) and ParC (D86N, S87C, I, K, N, R or Y; S88A or P, E91A, G, K or Q) [71,109,110] (Figure 1).

The MIC of ciprofloxacin depends on the character and combination of substitutions [101]. There is a huge range of MICs described for a single mutation and combinations of them. Although the strains were tested in genetically related groups, they may differ in the presence of other resistance determinants not included in the study. A lack of substitution was present in 935 isolates of ST6962 and ST9363 (MLST), with the range of MICs from ≤0.25 to 32 mg/L, and most often, MICs ≤ 0.25 mg/L (99%). One substitution of ParC, S97R, occurred in ST13526 (MLST) and conditioned a range of MICs from ≤0.25 to 4 mg/L, and most often, an MIC ≤ 0.25 mg/L (95%). Two GyrA substitutions, S91F and D95A, occurred in ST1583 (MLST) and conditioned a range of MICs from ≤ 0.25 to 2 mg/L, and most often, the MIC = 1 mg/L (35%). Three substitutions, two in GyrA: S91F, D95A and one in ParC: S87N, were present in ST1588 (MLST) and conditioned a range of MICs from ≤0.25 to 4 mg/L, and most often, the MIC = 2 mg/L (44%). Three GyrA substitutions, S91F, D95A and ParC: D86N, were present in ST8143 and ST9365 (MLST) and conditioned a range of MICs from ≤0.25 to 16 mg/L, and most often, the MIC = 4 mg/L (39%) or the MIC = 8 mg/L (31%). Three GyrA substitutions, S91F, D95A and ParC: S87R, were present in ST13526, ST10314, ST8143 and ST7822 (MLST) and conditioned a range of MICs from ≤0.25 to 32 mg/L, and most often, the MIC = 4 mg/L (56%) or the MIC = 8 mg/L (37%). Three GyrA substitutions, S91F, D95G and ParC: S87R, were present in ST1901 and ST1579 (MLST) and conditioned a range of MICs from ≤0.25 to 32 mg/L, and most often, the MIC = 4 mg/L (42%). Three GyrA substitutions, S91F, D95G and ParC: E91G, were present in ST7363 (MLST) and conditioned a range of MICs from ≤0.25 to 32 mg/L, and most often, the MIC = 8 mg/L (41%). Three GyrA substitutions, S91F, D95G and ParC: D86N, occurred in ST7827 (MLST) and conditioned a range of MICs from ≤0.25 to 32 mg/L, and most often, the MIC = 8 mg/L (39%). Three substitutions of GyrA, S91F, D95N and ParC: S87I, occurred in ST7371 (MLST) and conditioned an MIC of 32 mg/L (100%) [101].

## 5. Resistance to Tetracyclines

Tetracyclines are antibiotics that inhibit protein synthesis by interfering with the 30S subunit of the ribosome (Figure 1). The criterion for tetracycline resistance according to the EUCAST is an MIC > 1.0 mg/L, and according to the CLSI, is an MIC ≥ 2.0 mg/L [11,12].

The TetM (ribosomal protection) protein is encoded by conjugative plasmids. Two variants of the *tetM* gene have been described, which have been named Dutch and American (MIC tetracycline 16–64 mg/L), and were found in 41,998–42,003 bp conjugative plasmids (GenBank acc. CP068762, CP045833). Two types of plasmids of 25.2 Mda have also been described, which have also been named Dutch (pOZ101; GenBank L12242) and American (pOZ100; GenBank L12241). In Poland, as in many other European countries, the Dutch TetM type was more common than the American type [111]. The most common correspondence is between gene type and plasmid type [112,113].

Other mechanisms determine the MIC of tetracycline 2–4 mg/L. Individually, the MIC can be >4 mg/L. Out of 1479 isolates, 8 such strains have been described [101]. These include mutations in the chromosomal gene *mtrR* and its promoter regions, causing overproduction of the membrane pump protein MtrCDE, and mutations in the genes *porB*, *pilQ* and *rpsJ* (V57M substitution in the ribosomal protein S10) [114] (Figure 1). However, alterations of the *pilQ* gene in *N. gonorrhoeae* are unlikely contributors to decreased susceptibility to tetracyclines in clinical gonococcal strains because the bacteria require an intact type IV pilus machinery for infection [77].

## 6. Resistance to Spectinomycin

Spectinomycin, an antibiotic classified as an aminocyclitol, inhibits protein translation by binding to 16S rRNA (helix 34; near base-paired nucleotides G1064-C1192) within the 30S subunit of the ribosome (Figure 1), and inhibits elongation factor G (EF-G) translocation, which is catalyzed by peptidyl-tRNA translocation from the A site to the P site during polypeptide elongation [115,116,117,118,119].

The criterion for spectinomycin resistance according to the EUCAST is an MIC > 64 mg/L, and according to the CLSI, is an MIC ≥ 128 mg/L [11,12]. The cause of *N. gonorrhoeae* resistance to spectinomycin may be due to a C1192U transition in 16SrRNA and mutations in the *rpsE* gene encoding the 5S ribosomal protein (deletion of codon 27 (valine) and a K28E substitution) [11].

## 7. Resistance to Gentamicin

Gentamycin is classified as an aminoglycoside. Aminoglycosides are bactericidal antibiotics that inhibit protein synthesis by interfering with the 30S subunit of the ribosome (Figure 1). There are no criteria for the resistance of *N. gonorrhoeae* to gentamicin according to the EUCAST and CLSI.

A missense mutation in the *fusA* gene, which encodes elongation factor G (EF-G) and causes an A563V substitution in the IV domain of EF-G, has been described (Figure 1). The mutant allele was named *fusA2*. Holley et al. showed that *fusA2* could increase the MIC of gentamicin 4-fold to an MIC = 32 mg/L. Although possession of *fusA2* impaired neither in vitro gonococcal growth nor protein synthesis, it caused a fitness defect during the experimental infection of the lower genital tract in female mice [120].

## 8. Other Drugs Active against *N. gonorrhoeae*

### 8.1. Zoliflodacin

Zoliflodacin (spiropyrimidinetrione gyrase inhibitor, ETX0914) is a 5,5′-pyrimidinetrione derivative with an adjacent benzisoxazole ring [121] (Figure 1). The drug is currently in phase III clinical trials. MICs of zoliflodacin were tested in 1209 *N. gonorrhoeae* strains isolated in 2018 in 25 EU/EEA countries. The MIC50, MIC90 and MIC range of zoliflodacin were 0.125, 0.125 and ≤0.004–0.5 mg/L, respectively. Resistance to ciprofloxacin, azithromycin, cefixime and ceftriaxone was 49.9%, 6.7%, 1.6% and 0.2%, respectively [122]. A study of 873 *N. gonorrhoeae* strains isolated in 21 European countries between 2012 and 2014 showed the MIC range, modal MIC, MIC50 and MIC90 were ≤0.002 to 0.25 mg/L, 0.125 mg/L, 0.064 mg/L and 0.125 mg/L, respectively [123]. Zoliflodacin showed no cross-resistance with the other antimicrobial agents tested. GyrB was highly conserved, and no *gyrB* mutations conditioning zoliflodacin resistance were found. None of the fluoroquinolone-target resistance mutations, GyrA or ParC, or mutations causing overexpression of the efflux pump MtrCDE had a significant effect on the MIC of zoliflodacin [122]. In a phase II clinical trial (RCT), a single 3 g dose of zoliflodacin resulted in a 100% cure rate for uncomplicated genitourinary (47/47) and rectal (6/6) gonorrhea, and the cure rate for pharyngeal gonorrhea was 78% (7/9). Zoliflodacin was well tolerated, with limited transient gastrointestinal side effects [124]. To date, no clinical isolates of zoliflodacin-resistant gonococci have been identified, but in vitro mutants with GyrB D429A/N or K450N/T substitutions have been selected that had zoliflodacin MICs of 1–2 mg/L [122,125].

### 8.2. Sitafloxacin

Sitafloxacin (DNA gyrase and topoisomerase IV inhibitor) belongs to a new generation of fluoroquinolones. The activity of sitafloxacin was studied against 250 strains of *N. gonorrhoeae* isolated between 1991 and 2013. Sitafloxacin had bactericidal activity, with MIC, MIC50 and MIC90 ranges of ≤0.001–1, 0.125 and 0.25 mg/L, respectively. There was a high correlation between the MICs of sitafloxacin and ciprofloxacin, but the MIC50 and MIC90 of sitafloxacin were 6-fold and >6-fold lower, respectively [126]. The MIC of sitafloxacin was tested in 35 ciprofloxacin-resistant *N. gonorrhoeae* isolates (ciprofloxacin MIC from 2 to 32 mg/L). The MIC of sitafloxacin ranged from 0.03 to 0.5 mg/L. None of the identified substitutions in the QRDR of GyrA and ParC increased the MIC of sitafloxacin above 0.5 mg/L [127].

### 8.3. Delafloxacin

Delafloxacin is an inhibitor of DNA gyrase and topoisomerase IV belonging to the new generation of fluoroquinolones. [128]. The activity of delafloxacin was tested against 117 strains of *N. gonorrhoeae* isolated between 2012 and 2015. The MIC50, MIC90 and MIC range of delafloxacin were 0.06 mg/L, 0.125 mg/L and ≤0.001 to 0.25 mg/L, respectively. The frequency of spontaneous mutation ranged from 10^−7^ to <10^−9^ [128]. The mutant had an S91Y substitution in GyrA and a delafloxacin MIC of 1 mg/L [128].

### 8.4. Gepotidacin

Gepotidacin (GSK2140944) is a novel, first-in-class triazaacenaphthylene, DNA gyrase and topoisomerase IV inhibitor [129,130,131] (Figure 1). Phase II clinical trials (RCTs) evaluating the use of gepotidacin in a single oral dose of 1.5 and 3 g for the treatment of uncomplicated gonorrhea showed 97% and 95% efficacy, respectively. The activity of gepotidacin against 252 strains of *N. gonorrhoeae* was tested. The modal MIC, MIC50, MIC90 and MIC range of gepotidacin were 0.5, 0.5, 1 and 0.032–4 mg/L, respectively [129]. Inactivation of the efflux pump MtrCDE was shown to lower the MIC of gepotidacin. A D86N substitution in ParC and A92T substitution in GyrA were found to be associated with high MIC values of gepotidacin [129]. In another study, the MIC50, MIC90 and MIC range of gepotidacin were 0.12–0.25, 0.5 and 0.06–1 mg/L, respectively. Two *N. gonorrhoeae* strains with MICs of 1 mg/L achieved treatment failure after a single dose of gepotidacin and the MICs of *N. gonorrhoeae* strains increased from 1 to ≥32 mg/L [131]. The PAEs (postantibiotic effects) for gepotidacin against the wild-type strain ranged from 0.5 to >2.5 h, and the PAE-SMEs (subinhibitory effects) were >2.5 h [130].

### 8.5. Solithromycin

Solithromycin (CEM-101) is a novel oral fluoroketolide antimicrobial with substantial in vitro activity against *N. gonorrhoeae*.

It also shows activity against *Chlamydia trachomatis* and *Mycoplasma genitalium*. Solithromycin binds to the 23S rRNA in the 50S subunit of the ribosome, which causes inhibition of bacterial protein synthesis [132,133] (Figure 1). The drug is currently in phase III clinical trials. Solithromycin displayed a MIC50 and MIC90 of 0.0625 and 0.125 mg/L for clinical strains of *N. gonorrhoeae* [134]. Two groups of gonorrhea patients were studied. One group received oral solithromycin 1000 mg and the other group received intramuscular ceftriaxone 500 mg plus oral azithromycin 1000 mg. Eradication of *N. gonorrhoeae* was achieved in 80% of patients receiving solithromycin and in 84% of patients receiving ceftriaxone plus azithromycin. The incidence of adverse events was higher in the solithromycin group than in the ceftriaxone plus azithromycin group (53% and 34%), most commonly, diarrhea (24% and 15%) and nausea (21% and 11%) [135].

### 8.6. Lefamulin

Lefamulin (BC 3781) is a new semisynthetic pleuromutilin. Lefamulin inhibits bacterial protein synthesis by interfering with the peptidyltransferase center in the 23S rRNA in the 50S subunit of the ribosome [136,137,138] (Figure 1). Modal MIC, MIC50 and MIC90 values and the MIC range of lefamulin are 0.5 mg/L, 0.25 mg/L, 1 mg/L and 0.004 to 2 mg/L, respectively. The efflux pump MtrCDE activity was shown to have a significant effect on the MIC values of lefamulin. Inactivation of the efflux pump MtrCDE reduced lefamulin MIC values by 15- to 60-fold in various *N. gonorrhoeae* strains while inactivation of the efflux pump MacAB and NorM did not change the lefamulin MIC [136].

### 8.7. Ertapenem

Ertapenem is a beta-lactam antibiotic from the carbapenem group and is used to treat infections with certain Gram-negative bacilli. MICs of ertapenem were tested in 259 *N. gonorrhoeae* isolated in 2013–19 in China. The MIC range was 0.006 mg/L to 0.38 mg/L, MIC50 was 0.032 mg/L and MIC90 was 0.125 mg/L. A total of 17.0% of isolates had MICs of ertapenem ≥0.125 mg/L, and 3.9% had MICs ≥ 0.25 mg/L. In nine isolates of *N. gonorrhoeae* resistant to oxyimino-cephalosporins (MIC ceftriaxone ≥ 0.5 mg/L and MIC cefixime ≥ 2 mg/L), the MIC range of ertapenem was 0.023 to 0.19 mg/L, MIC50 was 0.094 mg/L and MIC90 was 0.19 mg/L. The MIC50 and MIC90 values of ertapenem increased from 0.023 mg/L and 0.047 mg/L in 2013 to 0.047 mg/L and 0.125 mg/L in 2019, respectively [26].

### 8.8. Modithromycin and EDP 322

Modithromycin (6,11-bridged bicyclolides, EDP-420, EP-013420, S-013420) and EDP-322 are new macrolide drugs in clinical trials. [139]. Modithromycin interferes with the II and V domains of the 23S rRNA in the 50S subunit of the ribosome, which leads to inhibition of protein synthesis [139]. The sensitivity of 250 *N. gonorrhoeae* strains to modithromycin and EDP-322 was determined. The MIC range, modal MIC, MIC50 and MIC90 for modithromycin and EDP-322 were 0.004-256, 0.25, 0.25 and 1 mg/L and 0.008-16, 0.5, 0.5 and 1 mg/L, respectively. The activity of modithromycin and EDP-322 was higher than that of azithromycin [139].

### 8.9. Aminoethyl Spectinomycins

A series of aminoethyl spectinomycins (AmSPCs) obtained by modifying the 3′ keto group in the C ring of spectinomycin were studied. AmSPC preparations were shown to have slightly higher in vitro activity against *N. gonorrhoeae* and *Chlamydiatrachomatis* than spectinomycins. *N. gonorrhoeae* strains resistant to spectinomycin were also resistant to AmSPC [140].

### 8.10. Fosfomycin

The fosfomycin phosphoenolpyruvate analogue antibiotic is used in the treatment of cystitis. It acts as a bactericide by binding to the enolpyruvate transferase (MurA), and consequently, inhibiting cell wall synthesis (Figure 1). The susceptibility of 89 *N. gonorrhoeae* isolates to fosfomycin was tested. The MIC50, MIC90 and MIC range were 8 mg/L, 16 mg/L and ≤1 to 32 mg/L, respectively [141].

### 8.11. TP0480066 and Other Antimicrobials

TP0480066 is a novel 8-(methylamino)-2-oxo-1,2-dihydroquinoline (MAOQ) derivative (DNA gyrase and topoisomerase IV inhibitor). The TP0480066 MIC of fourteen *N. gonorrhoeae* reference strains (including strains resistant to ciprofloxacin and levofloxacin, as well as to other drug groups) was determined. The MIC range obtained for these strains was from ≤0.00012 to 0.0005 mg/L [142].

The activity (MIC 0.125 mg/L) of the newly synthesized compounds N-(1,3,4-oxadiazol-2-yl) benzamides (HSGN-237 and -HSGN-238) was also demonstrated against a strain of *N. gonorrhoeae* with a high resistance to azithromycin [143].

## 9. Epidemiological Typing of *N. gonorrhoeae*

Closely related to the acquisition and presence of resistance genes in *N. gonorrhoeae* is molecular typing, a fundamental method in the study of many bacterial species. Molecular typing makes it possible to trace the occurrence, spread and evolution of multidrug-resistant strains globally. Various molecular typing systems exist. In *N. gonorrhoeae*, in some typing systems (e.g., NG-STAR), sequence types are determined solely on the basis of mutations in genes associated with resistance or reduced sensitivity to beta-lactams, macrolides and fluoroquinolones.

The typing of *N. gonorrhoeae* is usually carried out based on four methods related to the sequencing of the whole genome or its fragments: WGS (whole-genome sequencing), NG-MAST (*Neisseria gonorrhoeae* multi-antigen sequence typing), MLST (multi-locus sequence typing) and NG-STAR (*Neisseria gonorrhoeae* sequence typing for antimicrobial resistance).

### 9.1. Whole-Genome Sequencing (WGS)

The WGS method makes it possible to divide the population of strains under study into related groups which we call clades. Initially, the WGS method played primarily an auxiliary role, allowing us to determine the sequence types of *N. gonorrhoeae* NG-MAST, MLST and NG-STAR strains under study. By the end of 2022, a global database of *N. gonorrhoeae* clades had not been created, making it impossible to compare strains with a broader perspective. Harisson et al. [49] divided the studied pool of *N. gonorrhoeae* strains based on WGS into eight clusters. Clusters 1–4 included strains ST1901 (MLST), cluster 5 (ST1588), cluster 6 (ST1893), cluster 7 (ST9363) and cluster 8 (ST1580). Reimche et al. [101] divided the 1479 *N. gonorrhoeae* strains isolated in the US in 2018 into 23 major clusters and into 2 lineages, A and B. Lineage A (76%) had elevated MICs for multiple antibiotics and lineage B (24%) was mostly antibiotic sensitive. The three largest were cluster 16 (278 isolates (19%); MLSTs: ST9363, ST11422, ST11428, ST8134), cluster 17 (159 isolates (11%); MLST: ST81433, ST13526) and cluster 10 (96 isolates (6.5%); MLST: ST10314, ST7822, ST1901) [101].

### 9.2. Neisseria gonorrhoeae Multi-Antigen Sequence Typing (NG-MAST)

The NG-MAST method has been the most widely used method for typing *N. gonorrhoeae* since 2004. It involves sequencing a 490 bp fragment of the *porB* gene (430–511 bp as of mid-2021), encoding porin B and a 390 bp fragment of the *tbpB* gene (367–416 bp as of mid-2021) and encoding the B subunit of the transferrin-binding protein. The allele number of each gene under study is being determined in the worldwide NG-MAST database [144]. By the end of 2020, about 12,800 alleles of the *porB* gene and about 3200 alleles of the *tbpB* gene had been described. [71]. In mid-2021, the NG-MAST database was reorganized (current name NG-MASTv2.0). *porB* sequences with a number greater than 11,028 and *tbpB* sequences with a number greater than 2868 were removed. Currently (20 July 2022), the database contains 11911 *porB* alleles (numbering from 1 to 12059) and 2994 *tbpB* alleles (numbering from 1 to 3087). Based on the combination of *porB* and *tbpB* alleles, the NG-MAST sequence type is determined. By the end of 2020, about 22,000 NG-MAST sequence types had been described [71]. As a result of the reorganization of the database in mid-2021, sequence types with numbers higher than 11028 were removed. About 100 removed STs were re-entered into the database, but under different numbers. Currently (20 July 2022), the base contains 20218 STs (numbering from 1 to 20,729). Using the WGS method, NGMASTER is a tool for rapidly determining NG-MAST types in silico from *N. gonorrhoeae* genomes [145]. Sequenced NG-MAST types are grouped within several hundred genogroups. Genogroup membership is determined by comparing the *porB* and *tbpB* sequences of the sequence type from which the genogroup is named (e.g., ST-A) with the *porB* and *tbpB* sequences of another ST (e.g., ST-B and ST-C). If *porB* differs by ≤5 bp and *tbpB* is the same, *tbpB* differs by ≤4 bp and *porB* is the same, or the sum of differences in *porB* and *tbpB* in ST-A and ST-B and ST-A and ST-C is ≤5 bp (substitutions, insertions, deletions, inversions are treated as a change of 1 bp), then we can include ST-B and ST-C in genogroup GST-A. From this definition, it follows that between ST-B and ST-C, they can differ by 1 to 10 bp. The choice of genogroup name (traditional or referring to the most common ST in a particular area or time period) is also debatable. The largest genogroup, G1407, includes more than 500 sequence types, and each of the other large genogroups such as G21, G51, G225, G2992, G4822 and G10799 contains more than 200 sequence types. Among the *N. gonorrhoeae* isolated in 21 EU countries in 2013 and characterized by the Euro-GASP, the most frequently isolated were ST1407 (7.6%), ST2992 (6.6%), ST2400 (3.9%), ST4995 (3. 0%) and ST21 (2.4%). The most frequently isolated genogroups were G1407 (14.8%), G2992 (7.7%), G21 (6.2%), G2400 (5.6%), G51 (5.1%), G225 (4.0%), G4995 (3.2%) and G387 (2.5%) [146]. Among the *N. gonorrhoeae* isolated in 26 EU countries and the UK in 2018 and characterized by the Euro-GASP, the most frequently isolated were ST11461 (4.7%), ST5441(3.7%), ST12302 (3.4%), ST14994 (3.2%) and 14769 (3.0%) [27]. The most frequently isolated NG-MAST genogroups in the EU in 2018 were: G12302 (5.6%), G5441 (5.6%) and G11461 (5.4%). The incidence of the G1407 genogroup, which was dominant in the EU in 2009–10 (23.3%) and 2013 (16.5%), decreased to 2.1% in 2018. G12302 is most often NG-STAR CC168 and CC63, and MLST ST9363. G5441 is most often NG-STAR CC442. G11461 is most often NG-STAR CC42 [27]. The most common NG-MAST sequence types found in selected countries are shown in Table 5 [26,27,42,50,51,52,101,146,147,148,149,150,151].

### 9.3. Multi-Locus Sequence Typing (MLST)

The MLST method has long been used to type many bacterial and some fungal species. Typing of *N. gonorrhoeae* involves sequencing fragments of 450–500 bp of seven basal metabolism genes: *abcZ* (putative ABC transporter), *adk* (adenylate kinase), *aroE* (shikimate dehydrogenase), *fumC* (fumarate hydratase), *gdh* (glucose-6-phosphate dehydrogenase), *pdhC* (pyruvate dehydrogenase subunit) and *pgm* (phosphoglucomutase). In the publicly available worldwide PubMLST database of *Neisseria* spp., the allele number of each gene under study is determined, and based on the combination of the seven alleles, the MLST sequence type is determined [78,152]. The database contains only 1264 MLST sequence types of *N. gonorrhoeae* (numbering from ST1024 to ST16767 with breaks), which indicates a relatively low degree of differentiation of *N. gonorrhoeae* strains. The method performs much better for other *Neisseria* species. The most common MLST *N. gonorrhoeae* sequence types isolated were ST1901, ST7363, ST7822, ST7827, ST9362, ST9363, ST10314, ST11429, ST11463, ST11516, ST11864 and ST11990. In EU countries, the most common MLST *N. gonorrhoeae* sequence types are ST1901, ST9363 and ST7363. Among *N. gonorrhoeae* isolated in 26 EU countries in 2018, ST9363 was the most common (9.3%) [27]. Among the 1479 *N. gonorrhoeae* strains isolated in the US in 2018, ST9363 (10.7%), ST8143 (6.4%), ST1584 (4%), ST7363 (3.9%) and ST1599 (3.8%) were the most frequently isolated [101].

### 9.4. Neisseria gonorrhoeae Sequence Typing for Antimicrobial Resistance (NG-STAR)

The NG-STAR method, used since 2017 [64,65], involves sequencing fragments of seven genes of *N. gonorrhoeae*: *penA* (encodes PBP2; 524 alleles), *mtrR* (encodes membrane pump repressor protein *mtrCDE*; 515 alleles), *porB* (encodes porin B; 64 alleles), *ponA* (encodes PBP1; 19 alleles, numbered 1–18 and 100), *gyrA* (encodes gyrase subunit; 58 alleles), *parC* (encodes topoisomerase IV subunit; 175 alleles) and *23S rRNA* (contains target site for azithromycin; 71 alleles). Some mutations in the above-mentioned genes condition resistance to beta-lactam antibiotics, fluoroquinolones and macrolides. It is unfortunate that in the case of mutations in the 23S rRNA, no account was taken of how many alleles of the gene (the *23S rRNA* gene always occurs in four copies) the mutation occurs in, which would translate directly into azithromycin resistance or lack thereof.

There are two publicly available global databases: the Canadian NG-STAR v. 2.0 [153] and the European Pub MLST NG-STAR [64]. Based on the sequence (usually using the WGS method), the allele number of each gene under study is determined, and based on the combination of the seven alleles, the NG-STAR sequence type is determined. As of May 2022, 4340 ST NG-STAR types have been described (numbering up to ST4464). Among the *N. gonorrhoeae* isolated in 26 EU countries in 2018 and surveyed by the Euro-GASP, the most frequently isolated NG-STAR type was ST442 [18]. Among the 1479 *N. gonorrhoeae* isolated in the US in 2018, the most frequently isolated NG-STAR type was ST436 (6%), ST63 (4.9%) and ST520 (3.9%) [101]. In 2021, Golparian et al. [39] divided 1-2602 ST NG-MAST into 317 clonal complexes (CC) and 169 sequence types that (ST) NG-MAST defined as ungroupable.

## 10. Conclusions

The most serious problem regarding *N. gonorrhoeae* drug resistance is the emergence and spread of clones resistant to ceftriaxone and azithromycin. Many strains of *N. gonorrhoeae* with an MIC ≥ 0.5 mg/L of ceftriaxone have been described, but most of them have not spread worldwide. The first such clone, FC428, was described in Japan in 2015 and has spread in Asian countries, but is not observed in Europe and the US. A second possible option for acquisition of ceftriaxone resistance is the emergence and spread of extended-spectrum TEM beta-lactamase variants (many such variants have been described in Gram-negative bacilli, but the spectrum of none incules carbapenems). This event is also more likely in Asian countries where there is a high percentage of beta-lactamase production by *N. gonorrhoeae*. Additionally, of concern is the increase in the number of azithromycin-resistant *N. gonorrhoeae* strains observed in Europe in 2016–19. In light of these risks, it is expedient to search for new drugs active against *N. gonorrhoeae*.

## Figures and Tables

**Table 1:** *N. gonorrhoeae* strains with ceftriaxone MIC ≥ 0.5 mg/L.

Country	Year	Strain	CRO MIC mg/L	ST NG-STAR (CC)	PenA	PorB G120/A121	*mtrR*/MtrR	ST NG-MAST (GST)	ST MLST
China	2020	YL201 *	0.75	2238 (NA)	60.001	K/D	−35A Del	new	1600
Vietnam	2019–20	VN128T	0.5	233	60.001	K/D	−35A Del	7237 (G431)	13,871
Vietnam	2019–20	VN20T	0.75	233	60.001	K/D	−35A Del	1086 (G431)	13,871
Singapore	2019–20	18DG342	1	233	60.001	K/D	−35A Del	1086 (G431)	13,871
China	2019	SRRSH214	1	2208 (NA)	60.001	K/D	−35A Del	19,972 ^a^	1600
China	2019	SRRSH229	1	2208 (NA)	60.001	K/D	−35A Del	19972 ^a^	1600
China	2019	DG19112	0.5	233 (199)	60.001	K/D	−35A Del	20408 ^a^	13943
China	2019	SS74	0.5	new	60.001	NA	−35A Del	new	1903
China	2018	ZH545	0.5	2239 (NA)	60.001	D/wt	−35A Del	13272 (G13272)	7365
China	2018	GC250 *	0.5	1143 (199)	60.001	K/G	−35A Del	new	7365
China	2018	SC18-25	≥0.5	233 (199)	60.001	K/D	−35A Del	new	1903
China	2018	SC18-26	≥0.5	233 (199)	60.001	K/D	−35A Del	new	1903
China	2018	SC18-68	≥0.5	233 (199)	60.001	K/D	−35A Del	16059 (G564)	7363
China	2018	DG18193	0.5	new	60.001	NA	−35A Del	new	1903
China	2018	MM14	0.5	233 (199)	60.001	K/D	−35A Del	1086 (G359)	1903
Canada	2018	51742	0.5	233 (199)	60.001	K/D	−35A Del	3435 (G564)	1903
Ireland	2018	IR72	0.5	1133 (199)	60.001	K/N	−35A Del	17842 (G564)	1903
Australia	2018	A2543 **	0.5	996 (73)	60.001	K/D	−35 Del; G45D	16848 (G1866)	12039
The UK	2018	G97687 *	0.5	996 (73)	60.001	K/D	−35 Del; G45D	16848 (G1866)	12039
The UK	2018	G7944 *	0.5	996 (73)	60.001	K/D	−35 Del; G45D	16848 (G1866)	12039
The UK	2018	H18-502	1.0	233 (199)	60.001	K/D	−35A Del	1614 (G5267)	1903
The UK	2018	H18-209	1.0	233 (199)	60.001	K/D	−35A Del	1614 (G5267)	1903
China	2017	GC185	1	1143 (199)	60.001	K/G	−35A Del	new	1903
China	2017	SZ2017191	0.5	233 (199)	60.001	K/D	−35A Del	15240 (G564)	1903
France	2017	F90	0.5	133 (38)	54.00	K/D	−35 Del; G45D	3435 (G564)	1903
Denmark	2017	GK124	0.5	233 (199)	60.001	K/D	−35A Del	1614 (G5267)	1903
Canada	2017	47707	1.0	233 (199)	60.001	K/D	−35A Del	1614 (G5267)	1903
Australia	2017	A7536	0.5	233 (199)	60.001	K/D	−35A Del	15925 (G11110)	1903
Australia	2017	A7846	0.5	233 (199)	60.001	K/D	−35A Del	1614 (G5267)	1903
Japan	2017	KM383	0.5	233 (199)	60.001	K/D	−35A Del	16186 (G431)	1903
Japan	2017	KU17039	0.5	233 (199)	60.001	K/D	−35A Del	16186 (G431)	1903
Japan	2016	KU16054	0.5	233 (199)	60.001	K/D	−35A Del	3435 (G564)	1903
China	2016	BJ16148	0.5	233 (199)	60.001	K/D	−35A Del	3435 (G564)	1903
Japan	2015	FC498	0.75	233 (199)	60.001	K/D	−35A Del	3435 (G564)	1903
Japan	2015	FC428	0.5	233 (199)	60.001	K/D	−35A Del	3435 (G564)	1903
Japan	2015	FC460	0.5	233 (199)	60.001	K/D	−35A Del	3435 (G564)	1903
Argentina	2014	CCETS-7069	0.5	139 (139)	9.001	K/D	−35A Del	13064 (G21)	13637
Japan	2013	Tum15748	0.5	NA	169.001	NA	ND	6771 (G9909)	7359
Australia	2013	A8806; WHO-Z	0.5	227 (348)	64.001	K/D	wt	4015 (G11018)	7363
China	2012/13	GD4	0.5	NA	II	NA	ND	10208 (G5062)	NA
China	2012/13	HN9	0.5	NA	XXI	NA	ND	5913 (G1791)	NA
Spain	2012	F89; WHO-Y	1.0	16 (90)	34.001	K/N	−35A Del	1407 (G1407)	1901
France	2010	F89; WHO-Y	1.0	16 (90)	42.001	K/N	−35A Del	1407 (G1407)	1901
Japan	2009	HO41; WHO-X	2.0	226 (348)	37.001	K/D	−35A Del	4220 (G4019)	7363
China	2007	NA	0.5	NA	XVII	NA	ND	2288 (G1791)	NA

* Strain resistant to azithromycin, ** strain resistant to high concentration of azithromycin MIC ≥ 256 mg/L; ND, no data; CRO, ceftriaxone; CC, clonal complex; GST, genogroup; ^a^ sequence type (ST) removed from NG-MAST database in 2021.

**Table 2:** The effect of mutations in the *rpoB* and *rpoD* genes on changes in ceftriaxone and cefixime MICs.

Strain	RpoB	RpoD	PenA *	PorB G120/A121	PonA	Ceftriaxone MIC mg/L	Cefixime MIC mg/L
GCGS0364	wt	wt	9.001	K/D	L421P	0.023	NA
GCGS0364	G158V	wt	9.001	K/D	L421P	0.5	NA
GCGS0364	P157L	wt	9.001	K/D	L421P	0.75	NA
GCGS0457	wt	wt	9.001	G/V	L421P	0.012	0.016
GCGS0457	R201H	wt	9.001	G/V	L421P	0.19	>0.5
GCGS0457	wt	E98K	9.001	G/V	L421P	0.125	0.5
GCGS0457	wt	92–95 del	9.001	G/V	L421P	0.19	0.5

* NG-STAR PenA allele; wt, wild-type; NA, not applicable.

**Table 3:** Nonmosaic-type PenA.

PenA	Substitution *	PenA	Substitution *	PenA	Substitution *
I (18)		88 (1)	A501V, A516G	170 (2)	-
II (56) ^abc^	F504L, A516G	90 (1)	A516G	174 (1)	A516G
III (4)	A516G	94 (1)	N512Y	175 (1)	A516G
IV (1)	A516G, G542S	95 (1)	-	176 (1)	-
V (15) ^bc^	F504L, A516G, G542S	96 (1)	-	178 (1)	N512Y
VII (1) ^c^	A501V, A516G, G542S	97 (1)	-	179 (1)	-
IX (11) ^abc^	A516G	98 (2)	-	181 (1)	A501V, A516G, G542S
XI (2)	A501V, A516G	99 (1)	A311V	182 (1)	N512Y
XII (10) ^bc^	F504L, A516G	100 (15)	wt	183 (1)	-
XIII (9) ^bc^ 13.008	A501V, A516G A201V	102 (1)	A516G	184 (1)	-
XIV (19)	A516G	103 (6) 103.006	A501V, A516G A516G	185 (1)	-
XV (10)	-	104 (1)	A501T, A516G, G542S	186 (1)	-
XVI (1)	A516G	106 (1)	A516G, G542S	187 (1)	-
XVII (1) ^abc^	A501V, A516G, G542S	107 (1) ^c^	A501T, A516G, G542S	189 (1)	A516G, N542S
XVIII (5) ^bc^ 18.005	A501V, A516G, G542S A516G, G542S	109 (1) ^c^	A501V, A516G	190 (1)	A516G
XIX (18)	A516G	119 (1)	-	191 (1)	A516G
XXI (4) ^abc^	A501V, A516G	120 (1)	A501V, A516G	192 (1)	A516G
XXII (16)	F504L, A516G	122 (1)	A501V, A516G	193 (1)	A516G
40 (1)	-	123 (1)	-	194 (1)	A516G
41 (2)	A516G, G542S	125 (1)	A501T, A516G, G542S	197 (1)	A501P, A516G
43 (7) ^c^	A501V, A516G	126 (1)	A501T, A516G, G542S	199 (1)	A516G
44 (7)	A501T, A516G	127 (1)	N512Y	200 (1)	A516G
45 (3)	A516G	132 (1)	A516G	201 (1)	A516G
46 (1) ^c^	A516G	137 (1)	A516G, G542S	202 (1)	A311V, A516G
48 (2)	A516G	141 (1)	A501V, A516G	203 (1)	A516G
49 (1)	A501T, A516G	142 (1)	A501V, A516G	204 (1)	A516G
50 (2) ^c^	A516G	143 (1)	A501V, A516G	208 (1)	A516G
54 (3)	A501V, A516G	144 (1)	A501V, A516G	209 (1)	A501P, A516G
56 (1)	A501V, A516G	146 (1)	A501T, A516G, G542S	210 (1)	A516G
57 (1)	A501V, A516G	147 (1)	A501V, A516G	213 (1)	A501P, A516G
61 (1)	A516G	148 (1) ^bc^	A516G	218 (2)	A516G
66 (1)	N512Y	151 (1)	A516G	220 (1)	-
68 (1) ^c^	A516G	153 (1)	A516G	221 (1)	A516G
69 (5)	A516G	154 (1)	A501V A516G	222 (1)	A516G
70 (1)	A516G	155 (1)	A501V A516G	224 (1)	A516G
76 (3)	-	156 (1)	A311V A516G	225 (1)	A516G
77 (1)	A501T, A516G	157 (1)	A516G	226 (1)	A516G, G542S
79 (2)	-	158 (1)	A501V A516G	227 (1)	A516G, G542S
82 (1)	-	159 (1)	A516G	228 (1)	A516G
83 (1)	A516G	160 (1)	A516G	229 (1)	A516G
84 (1)	-	161 (1)	A516G	230 (1)	
86 (1)	A516G	162 (1)	A516G	231 (1)	A501V, A516G
87 (2)	A501T, A516G	163 (1)	A516G G542S	234 (1)	

Strains with ceftriaxone MICs of: ^a^ ≥0.5 mg/L, ^b^ 0.25 mg/L, ^c^ 0.125 mg/L; PenA = PBP2 was described, with the number of subtypes in parentheses; * selected substitutions in PenA alleles.

**Table 4:** Mosaic- and semimosaic-type PenA.

PenA	Substitution *	PenA	Substitution *	PenA	Substitution *
Mosaic-type PenA					
X (12) ^c^	V316T, F504L, N512Y	85 (1)	A501V, N512Y	166 (1)	N512Y
XXVII (4) ^c^	N512Y	92 (1)	N512Y	168 (1)	A501V, N512Y
XXXIV (27) ^abc^	N512Y	101 (1)	A501V	169 (1) ^a^	A311V, N512Y
XXXV (3)		105 (1)	N512Y	171 (1)	N512Y
37 (1) ^abc^	A311V, N512Y	108 (1)	N512Y	172 (1)	
38(1) ^abc^		110 (1)	N512Y	180 (1)	N512Y
42 (1) ^abc^	A501P, N512Y	111 (1)	N512Y	188 (1)	N512Y
51 (1) ^c^	N512Y	115 (1)		195 (1)	A311V, N512Y
52 (2)	N512Y	117 (1)	N512Y	196 (1)	N512Y
53 (3) ^c^	N512Y	118 (1)	N512Y	198 (1)	N512Y
55 (1) ^c^	N512Y	121 (2)	A311V A516G	205 (1)	N512Y
58 (1)	N512Y	124 (1)	A311V, N512Y	206 (1)	N512Y
59 (1) ^abc^	A311V, N512Y	129 (1)	A516G	207 (1)	A501P, A516G
60 (1) ^abc^	A311V, N512Y	131 (1)		211 (1)	N512Y
62 (1)	N512Y	133 (1)	N512Y	212 (1)	N512Y
63 (1)		134 (1)	A516G	214 (1)	A311V, N512Y
64 (1) ^abc^	A311V, N512Y	136 (1)	N512Y	215 (1)	A516G, G542S
67 (3)	N512Y	138 (1)	N512Y	217 (1)	N512Y
71 (1) ^c^	N512Y	139 (1)	Mosaic wild-type	219 (1)	N512Y
72 (1)	N512Y	145 (1)	N512Y	223 (1)	N512Y
74 (1)	N512Y	149 (1)	A501V	232 (1)	A311V, N512Y
75 (1)		152 (1)	N512Y	233 (1)	
Semimosaic-type PenA					
39 (1) ^c^		93 (1)		150 (1)	A516G G542S
47 (1)		112 (1)	N512Y	164 (1)	N512Y
65 (1)		113 (1)	N512Y	165 (1)	N512Y
73 (1)	N512Y	114 (1)		167 (1)	N512Y
78 (2)	A501T, N512Y	116 (1)		173 (1)	
80 (2)	A516G	128 (1)	A501V, A516G	177 (1)	
81 (1)	N512Y	130 (1)	N512Y	216 (1)	N512Y
89 (1)		135 (1)	N512Y		
91 (1)	N512Y	140 (1)	A516G		

Strains were described with ceftriaxone MIC: ^a^ ≥0.5 mg/L, ^b^ 0.25 mg/L, ^c^ 0.125 mg/L; (), number of subtypes; * selected substitutions in PenA alleles.

**Table 5:** Prevalence of NG-MAST sequence types in Europe and selected non-European countries.

Country	Year	n	NG-MAST ST (Genogroup)	% ST
Austria	2013 2018	54 183	3785 (G3785)/11575 (G11575)/4995 (G4995)/387 (G387)/225 (G225) 12302 (G4822)/387 (G387)/11461 (G11461)	16.7/11.1/7.4/7.4/7.4 11.5/5.5/4.4
Belgium	2013 2018	55 76	1407 (G1407)/387 (G387)/2992 (G2992) 5441 (G5441)/1513 (G1407)/2992 (G2992)	16.4/14.6/14.6 5.3/4/4
Denmark	2013 2018	56 99	1993 (G1993)/1407 (G1407)/2400 (G2400) 1993 (G1993)/11461 (G11461)/5441 (G5441)	17.9/12.5/7.1 31.3/5.1/4
France	2013 2018	58 37	645 (G645)/11352 (G11352)/225 (G225)/2400 (G2400)/2992 (G2992) 14769 (G14769)/15589 (G15589)/2 (G2)	8.6/5.2/3.5/3.5/3.5 10.8/8.1/5.4
Germany	2013 2018	50 114	4995 (G4995)/25 (G51)/359 (G359)/5441 (G5441)/9500 (G9500) 10386 (G11352)/15589 (G15589)/387 (G387)	8.3/6.3/6.3/6.3/6.3 5.3/5.3/4.4
Greece	2013 2018	50 79	3128 (G1407)/225 (G225)/4730 (G4730)/11055 (G225) 14994 (G14994)/3128 (G1407)/7445 (G7445)	18.8/10.4/10.4/10.4 10.1/6.3/6.3
Hungary	2013 2018	48 89	1407 (G1407)/995 (G995)/387 (G387)/8115 (G2400)/11046 (G11046) 387 (G387)/11461 (G11461)/13113 (G387)	20.8/12.5/6.3/6.3/6.3 6.7/5.3/5.3
Ireland	2013 2018	45 169	2992 (G2992)/384 (G30)/21 (G21)/437 (G225)/10843 (G225) 14769 (G14769)/10386 (G11352)/14700 (G11089)	15.6/11.1/4.4/4.4/4.4 19.5/5.9/5.3
Italy	2013 2018	49 98	2992 (G2992)/6360 (G2400)/2400 (G2400)/1407 (G1407) 5441 (G5441)/10386 (G11352)/11461 (G11461)	18.4/12.2/12.2/10.2 8.2/7.1/5.1
The Netherlands	2013 2018	89 190	2992 (G2992)/2400 (G2400)/8919 (G8919) 11461 (G11461)/15589 (G15589)/14994 (G14994)	10.1/10.1/5.6 6.8/5.3/4.7
Norway	2013 2018	55 113	1407 (G1407)/4275 (G1407)/2400 (G2400) 14700 (G11089)/4186 (G9909)/3935 (G4822)	9.1/7.3/5.5 10.6/8/7.1
Poland	2012 2018	108 73	1407 (G1407)/8391 (G225)/1861 (G225)/2992 (G2992) 11461 (G11461)/14769 (G14769)/1407 (G1407)	43.3/7.4/4.1/4.1 25/9.4/6.3
Portugal	2013 2018	109 122	1407 (G1407)/7445 (G7445)/2 (G2) 645 (G645)/4261 (G4261)/5441 (G5441)	15.6/11.0/4.6 11.4/5.2/5.2
Slovakia	2013 2018	56 76	1407 (G1407)/359 (G359)/11042 (G51) 10800 (G51)/9918 (G9918)/13595 (G225)	14.3/14.3/12.5 31.6/5.3/5.3
Slovenia	2013 2018	55 104	21 (G21)/10801 (G10801) /10800 (G51)/10798 (G10798) 15589 (G15589)/11461 (G11461)/5441 (G5441)	12.7/12.7/9.1/9.1 22.1/10.6/3.9
Spain	2013 2018	119 173	1407 (G1407)/2992 (G2992)/21 (G21) 14994 (G14994)/4186 (G9909)/5743 (G387)	10.9/6.7/6.7 6.9/5.8/2.9
Sweden	2013 2018	50 199	5445 (G21)/7445 (G7445)7164 (G7164) 9909 (G9909)/225 (G225)/5441 (G5441)	10/6/6 8/7/6
The UK	2013 2018	127 207	2992 (G2992)/51 (G51)/4995 (G4995) 11461 (G11461)/5441 (G5441)/14769 (G14769)	10.2/10.2/9.5 6.3/5.8/4.8
Europe (Euro-GASP)	2013 2018	1189 2375	1407 (G1407)/2992 (G2992)/2400 (G2400) ST11461 (G11461)/ ST5441 (G5441)/ ST12302 (G4822),	7.6/6.6/3.9 4.7/3.7/3.4
Canada	2013 2018	1183 3379	2400 (G2400)/9663(G9663)/5985 (G1710) 12302 (G4822)/14994 (G14994)/5985 (G1710)	12.1/7.4/6.1 18.2/16.6/5.8
China	2012–13 2013–19	920 259	2318 (G11352)/1866 (G1866)/4846 (G1933) 5308 (G5308)/7554 (G5308)/3356 (G2160)/270 (G809)/4539 (G10799)	3.6/2.7/2.0 8.5/6.6/2.2/2.7/2.7
Russia	2013 2018	142 151	807 (G51)/1152 (G387)/5941 (G51) 228 (G228)/14942 (G14942)/807 (G51)	11.3/6.3/4.2 14.6/5.3/4.6
The US	2014–16 2015–17 2018	649 399 1479	3935 (G4822)/8241 (G4822)/1407 (G1407) 3935 (G4822)/3169 (G225)/7638 (G7638)/8241 (G4822) 9918 (G9918)/11461 (G11461)/3935 (G4822)	5.3/3.4/3.2 7.3/4.8/3.8/3.0 3.6/2.6/2.0

## Abbreviations

CLSI	Clinical and Laboratory Standards Institute
CDC	Centers for Disease Control and Prevention
ECDC	European Centre for Disease Prevention and Control
EUCAST	European Committee on Antimicrobial Susceptibility Testing
Euro-GASP	European Gonococcal Antimicrobial Surveillance Programme
MIC	minimum inhibitory concentration
MLST	multi-locus sequence typing
NG-MAST	*Neisseria gonorrhoeae* multi-antigen sequence typing
NG-STAR	*Neisseria gonorrhoeae* sequence typing for antimicrobial resistance
PBP	penicillin-binding protein
ST	sequence type
WGS	whole-genome sequencing
